# S100a9 Knockdown Decreases the Memory Impairment and the Neuropathology in Tg2576 Mice, AD Animal Model

**DOI:** 10.1371/journal.pone.0008840

**Published:** 2010-01-21

**Authors:** Tae-Young Ha, Keun-A Chang, Jeong a Kim, Hye-Sun Kim, Seonghan Kim, Young Hae Chong, Yoo-Hun Suh

**Affiliations:** 1 Department of Pharmacology, College of Medicine, National Creative Research Initiative Center for Alzheimer's Dementia and Neuroscience Research Institute, MRC, Seoul National University, Seoul, South Korea; 2 Department of Microbiology, School of Medicine, Ewha Womans University, Yangcheonku, Seoul, South Korea; UMR CNRS 5226 - Université Bordeaux 2, France

## Abstract

Inflammation, insoluble protein deposition and neuronal cell loss are important features in the Alzheimer's disease (AD) brain. To investigate the regulatory genes responsible for the neuropathology in AD, we performed microarray analysis with APP_V717I_-CT100 transgenic mice, an animal model of AD, and isolated the S100a9 gene, which encodes an inflammation-associated calcium binding protein. In another AD animal model, Tg2576 mouse brain, and in human AD brain, induction of S100a9 was confirmed. The endogenous expression of S100a9 was induced by treatment with Aβ or CT peptides in a microglia cell line, BV2 cells. In these cells, silencing study of S100a9 showed that the induction of S100a9 increased the intracellular calcium level and up-regulated the inflammatory cytokines (IL-1β and TNFα) and iNOS. S100a9 lentiviral short hairpin RNA (sh-S100a9) was injected into the hippocampus region of the brains of 13-month-old Tg2576 mice. At two months after injection, we found that knockdown of S100a9 expression had improved the cognition decline of Tg2576 mice in the water maze task, and had reduced amyloid plaque burden. These results suggest that S100a9 induced by Aβ or CT contributes to cause inflammation, which then affects the neuropathology including amyloid plaques burden and impairs cognitive function. Thus, the inhibition of S100a9 is a possible target for AD therapy.

## Introduction

Alzheimer's disease (AD) is characterized clinically by global cognitive dysfunction and neuropathologically by an age-dependent formation of β amyloid (Aβ)-containing senile plaques, usually surrounded by reactive astrocytes, activated microglia and dystrophic neurites, and intracellular neurofibrillary tangles (NFTs) [1], [2], [3].

Neuroinflammation is also one of the prevalent features of AD brains, along with insoluble protein deposition and neurodegeneration [4], [5]. To investigate the regulatory genes responsible for the neuroinflammation related to AD, we performed microarray analysis with APP_V717I_-CT100 Tg (CT-Tg) mice, an animal model of AD. CT-Tg mice over-express 100 amino acids of C-terminal fragment of amyloid precursor protein (CT) with London mutation, which leads to extensive neuronal degeneration in the hippocampal area [6], cognitive impairment [7] and destruction of long-term potentiation (LTP) [8]. Several genes were significantly upregulated in these mice, and we focused on the S100a9 gene, whose expression was markedly increased.

S100a9 is an inflammation-associated calcium binding protein belonging to the S100 family [9], [10]. Up-regulated S100a9 in reactive microglia [9], [11] activates the p38 mitogen-activated protein kinase (MAPK) cascade, NF-κB or calcium-dependent signal transduction [12] and is involved in the production of proinflammatory cytokines, the regulation of neurite extension, cell migration, and calcium homeostasis [13]. Neurological diseases such as cerebral ischemia [14], traumatic brain injury [15] and AD [16] have been reported to be associated with altered expression/function of the S100 family members [17]. Recently, S100a9 was found to be increased within neuritic plaques and reactive glia and was proposed to participate in the inflammation of the AD pathogenesis [16]. However, the detailed molecular mechanism of these pathological events remains unknown.

Here, we provide evidence that S100a9 gene is significantly up-regulated in the brains of AD animal models, Tg2576 and CT-Tg mice, and of human AD patients. CT as well as Aβ has been known to be involved in the pathogenesis of AD [18], [19], [20], [21]. Treatment with Aβ or CT peptides induced S100a9 in a dose-dependent manner. Our data show that S100a9 is involved in the production of inflammatory cytokines induced by Aβ or CT in BV2 cells. Moreover, data from knockdown experiments show that S100a9 might be responsible for the neurodegeneration and cognitive deficits in Tg2576 mice.

## Results

### S100a9 Is Induced in the Brains of Alzheimer's Disease Animal Models and Human AD Patients

**Figure 1:**
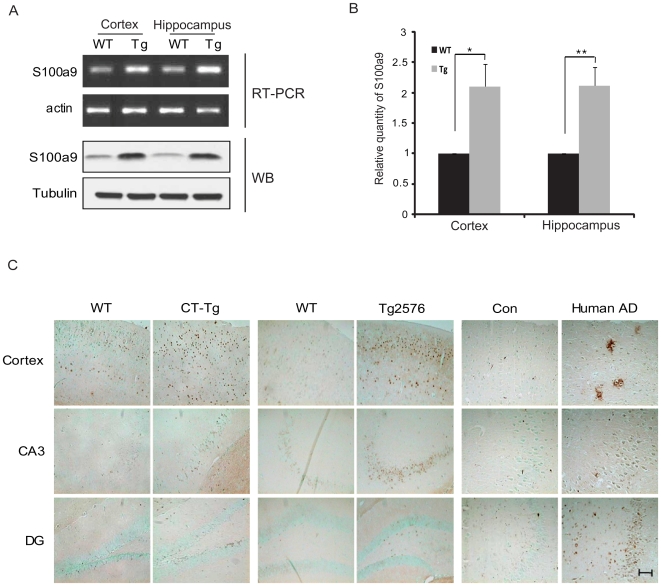
S100a9 was induced in the brains of CT-Tg, Tg2576 mice and Human AD patients. (A) mRNA levels of S100a9 were measured in the cortex and hippocampus of CT-Tg and age-matched WT mice brains by RT-PCR. Actin was used as a loading control. The protein level of S100a9 was checked by western blotting. Tubulin was used as a loading control. (B) S100a9 expression was normalized with that of tubulin for quantification (n = 5). Results are presented as the means ± SEM. *P <0.05, **P<0.01 by Student's *t-*test. (C) Immunoreactivities of S100a9 were examined in the cortex, hippocampus [CA3, dentate gyrus (DG)] of 11-month-old CT-Tg, of 11-month-old Tg2576 and of human AD brains compared with normal age-matched brains. Scale bar represents 100 µm.

To select candidate genes related to AD, microarray analysis was performed with the total RNA of the hippocampus of CT-Tg and age-matched wild-type (WT) mice. Some of the gene microarray results were validated by RT-PCR and western blot, and the S100a9 gene was selected for further studies. As seen in Figure 1A, mRNA levels of S100a9 were induced in the cortex and hippocampus of CT-Tg brains. On western blot and immunohistochemical analyses, S100a9 protein was significantly increased in the cortex and in the hippocampus of CT-Tg brains compared with that in region-matched WT brains (Figures 1A-C). In Tg2576 mice overexpressing Swedish type APP (sweAPP) [22], [23], S100a9 was also significantly increased in the cortex and hippocampal regions of the brain compared with that in WT brains (Figure 1C). To determine the physiological significance of the increased S100a9 levels in the brains of CT-Tg and Tg2576 mice, we examined the level of S100a9 in the brains of human AD patients and age-matched controls. AD brain sections (Figure 1C) and total lysates (Figures S1A&B) exhibited increased expression of S100a9 compared to control brains.

### Aβ or CT Induces S100a9 Expression in Microglia, BV2 Cells

**Figure 2:**
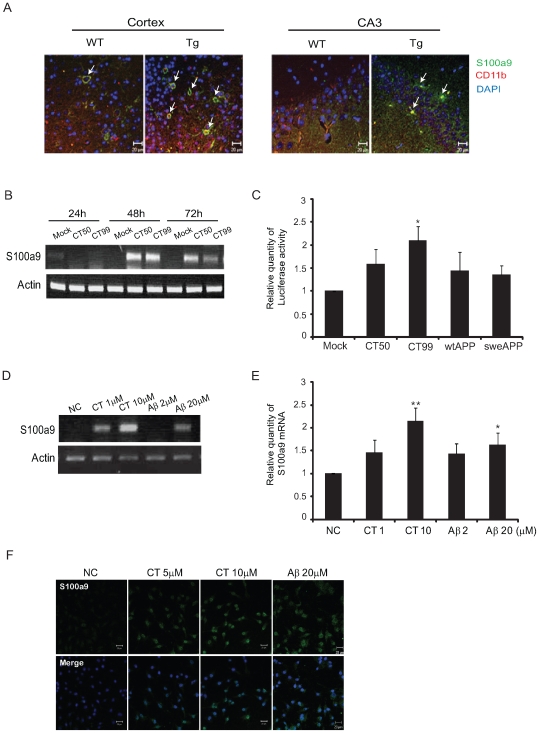
Aβ and CT induced S100a9 expression in BV2 cells. (A) Immunostaining for S100a9 (green) and CD11b (red) was performed in the cortex and CA3 of Tg and wild-type mouse brains. DAPI (blue) staining indicates the nuclei. Scale bars represent 20 µm. WT and Tg represent wild-type control and transgenic animals, respectively. (B) The mRNA levels of S100a9 were checked at 24 h, 48 h, and 72 h post-transfection with CT50 or CT99 by RT-PCR. (C) Human S100a9 promoter in pGL3 vector was cotransfected with CT, wtAPP or sweAPP in pcDNA vector and fe65 into SHSY5Y cells. 48 h after transfection, luciferase reporter assays were performed for CT50, CT99, wtAPP and sweAPP, the four S100a9 targets. Luciferase activities were normalized versus the obtained protein concentrations. The histogram shows the ratio of luciferase activity in transfected cells to empty vector-transfected cells (n = 5). (D) RT-PCR of S100a9 induced at 48 h post-treatment with 1 µM and 10 µM CT or 2 µM and 20 µM Aβ. (E) The quantification of RT-PCR normalized to actin (n = 8). (F) Immunocytochemistry; BV2 cells grown on a glass coverslip were treated with CT (5 µM and 10 µM) and Aβ (20 µM) for 48 h. S100a9-positive cells were visualized with FITC (green)-conjugated secondary antibodies. Nuclei were counter-stained with DAPI (blue). Scale bars represent 20 µm. All data are presented as the means±SEM. *P<0.05, **P<0.01 by Student's *t* –test.

Up-regulation of S100a9 expression was detected in the brains of CT-Tg and Tg2576 mice, as well as in AD brains, which might be related to over-produced Aβ and CT. In the brains of CT-Tg mice used in the microarray analysis, S100a9 was highly expressed in CD11b-positive microglia (Figure 2A), consistent with the report that S100a9 was up-regulated in reactive microglia [9]. To elucidate the pathological mechanism related to S100a9 in AD, we induced the endogenous expression of S100a9 in the microglia cell line, BV2 cells. After transfection with CT50 or CT99 in murine BV2 microglia cells, the gene expression of S100a9 was verified by RT-PCR. We found that the mRNA level of S100a9 was significantly increased in transfectants of CT50 or CT99 at 48 h and 72 h after transfection, especially at 48 h (Figure 2B). Aβ or CT could induce S100a9 via regulation of S100a9 promoter activity. We examined this possibility with a promoter activity assay. At 48 h post-transfection, the transactivational effect of CT50 and CT99 on S100a9 promoter activity was assessed (1.58±0.32 by CT50, 2.09±0.32 by CT99; Figure 2C). The effects of wild-type APP (wtAPP) and Swedish type APP (sweAPP) on S100a9 promoter activity were also measured (1.43±0.41 by wtAPP, or 1.35±0.20 by sweAPP), and especially, CT99 strongly increased S100a9 promoter activity (Figure 2C). Next, treatments with various concentrations of Aβ or CT peptides (1, 10 µM of CT or 2, 20 µM of Aβ) for 48 h induced mRNA level of S100a9 in a dose-dependent manner (Figure 2D). 10 µM CT significantly increased the mRNA level of S100a9 [ratio = 2.15±0.29, p = 0.0048 versus NC (negative control), student's *t*-test] (Figure 2E). Immunocytochemical analysis also showed that S100a9 was induced by Aβ or CT peptides in a dose-dependent manner (Figure 2F).

### S100a9 Induced by Aβ or CT Increases the Intracellular Calcium Level in BV2 Cells

As it has been reported that micromolar concentrations of S100 protein increase intracellular calcium ([Ca^2+^]_i_) levels [24], we hypothesized that CT induced the S100a9 gene, leading to increased [Ca^2+^]_i_ levels.

In BV2 cells treated with 0.1, 1 and 10 µM CT peptides or 1 and 10 µM Aβ peptides for 48 h, [Ca^2+^]_i_ levels were evaluated using the Fluo3/AM method. [Ca^2+^]_i_ levels were increased in a dose-dependent manner and their increases relative to control (ratio) were calculated (Figures S2A&B). 10 µM CT significantly increased the level of [Ca^2+^]_i_ (Figure S2B).

**Figure 3:**
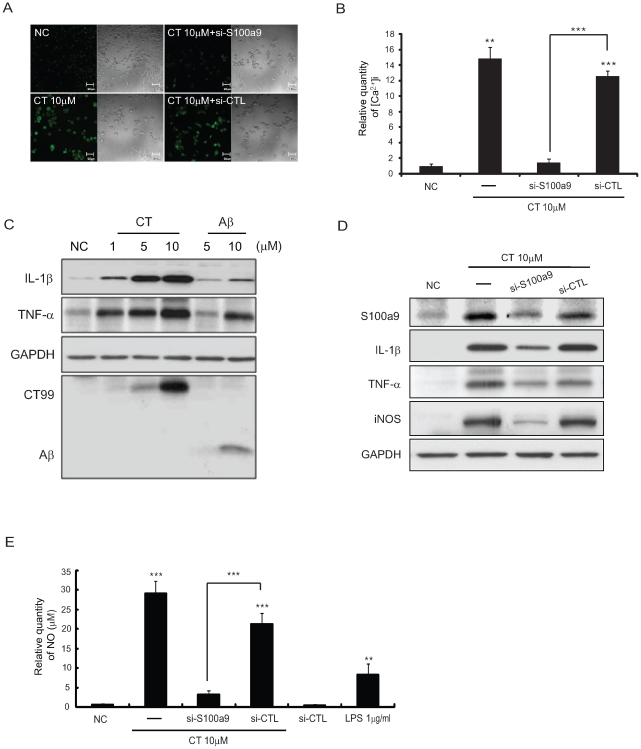
S100a9 induced by Aβ or CT increased [Ca^2+^]_i_ level and proinflammatory cytokines in BV2 cells. (A) [Ca^2+^]_i_ images obtained by Fluo3 AM at 48 h after treatment with 10****µM CT and 20 nM si-S100a9 in BV2 cells. Scale bars represent 50 µm. si-CTL is a control containing the scrambled sequence of S100a9 (B) The histogram shows the ratio of [Ca^2+^]_i_ levels to NC group. [Ca^2+^]_i_ levels in BV2 cells treated with CT and si-S100a9 was compared to that in BV2 cells with CT and si-CTL (control) (n = 5). (C) After treatment with 1, 5, or 10****µM CT and 5 or 10 µM Aβ to BV2 cells, the levels of the pro-inflammatory cytokines, IL-1β and TNF-α, were measured by western blotting. The membrane was stripped and reprobed with GAPDH to confirm equal loading and with 6E10 antibody to confirm intracellular CT and Aβ. (D) At 48 h post-treatment with si-S100a9, expressions of S100a9, IL-1β, TNF-α and iNOS were attenuated. The membrane was stripped and reprobed with GAPDH to confirm equal loading. (E) At 48 h post-treatment with 10****µM CT and si-S100a9, NO release (µM) was measured by Griess reagent. LPS (1 µg/ml) was used as a positive control. All data are presented as the means ± SEM (n = 10). NC: negative control (non-treated cell) **P<0.01, ***P <0.001 by Student's *t* -test and one-way ANOVA.

To determine whether S100a9 increased the [Ca^2+^]_i_ levels, we delivered S100a9 small interfering (si) RNA (si-S100a9) into BV2 cells treated with CT peptides. Knockdown of the S100a9 gene reduced the expression of S100a9 (Figure 3D) and significantly attenuated the increase of [Ca^2+^]_i_ levels by CT (from ratio = 12.63±0.65 to ratio = 1.49±0.45, p = 0.00016 versus si-CTL/CT 10 µM, Student's *t-* test). si-CTL did not affect the [Ca^2+^]_i_ level (Figures 3A&B).

We also investigated the levels of [Ca^2+^]_i_ by Aβ treatment in combination with si-S100a9 and knockdown of the S100a9 gene significantly attenuated the increase in [Ca^2+^]_i_ levels by 10 µM of Aβ (from ratio = 3.31±0.58 to ratio = 1.02±0.20, p = 0.0086 versus si-CTL/Aβ 10 µM, Student's *t -*test) (Figure S3).

### Proinflammatory Cytokines Are Increased in BV2 Cells Treated with Either Aβ or CT Peptides

The increase of [Ca^2+^]_i_ may initiate the inflammatory response in activated microglia [25]. Here, we found that either Aβ or CT treatment significantly increased the proinflammatory cytokines, IL-1β and TNF-α, in a dose-dependent manner (Figure 3C). To test the effects of S100a9 induction on the production of proinflammatory cytokines, S100a9 was silenced with si-S100a9. We found that the levels of IL-1β and TNF-α induced by CT were reduced by 60% with si-CTL, as was the expression of S100a9 (Figure 3D). The increased level of iNOS by CT was also reduced to about 70% by si-S100a9 (Figure 3D). As shown in Figure 3E, silencing of the S100a9 gene significantly attenuated CT-induced nitric oxide (NO) release (10 µM for 48 h) from 21.29±2.78 (µM) to 3.19±0.88 (µM).

### S100a9 Knockdown Attenuates Learning and Memory Impairment in Tg2576 Mice

**Figure 4:**
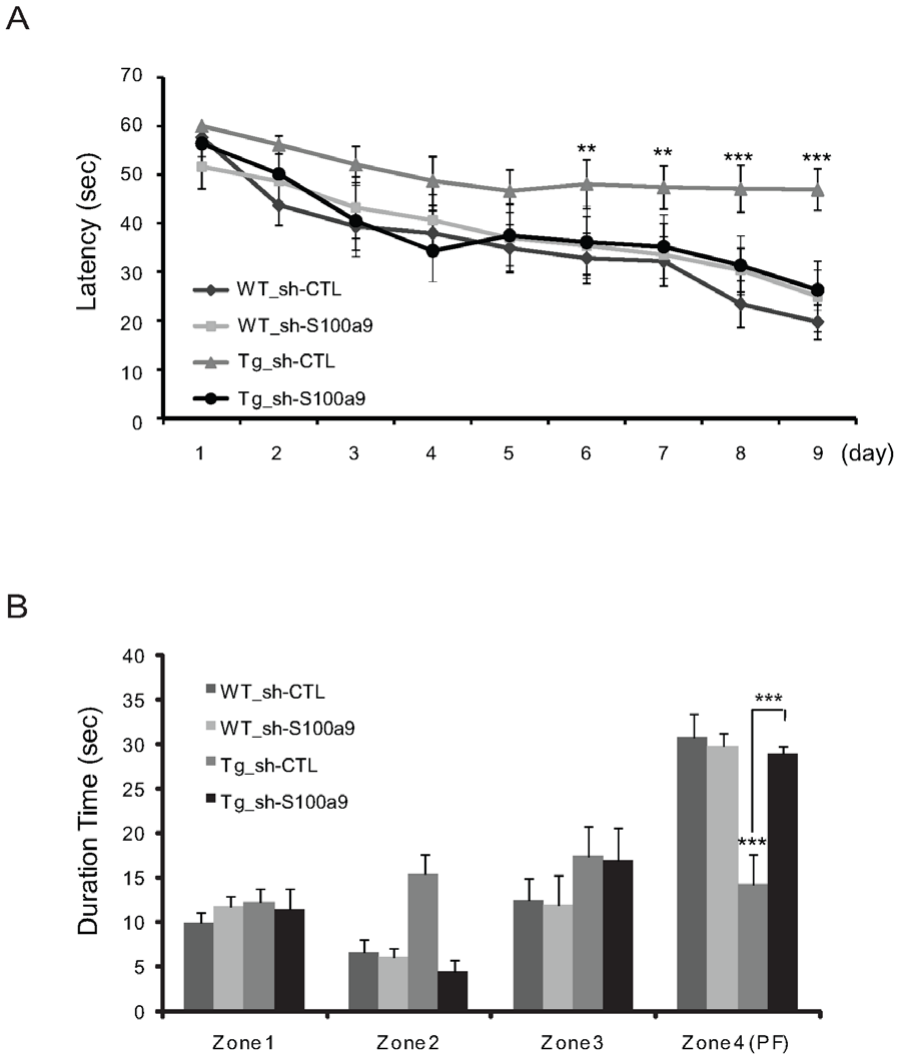
Treatment with shRNA-S100a9 attenuated learning and memory impairment in Tg2576 mice. (A) Morris water maze task was performed 2 months after lentiviral infection. The task was conducted for 9 consecutive days. A significant difference was observed between the Tg_sh-S100a9 group and the Tg_sh-CTL group from the 6^th^ day of the Morris water maze task. (B) The probe test was carried out 48 h after the final trial. Tg_sh-S100a9 group showed memory improvement compared to Tg_sh-CTL in zone 4 where the platform had been hidden (n = 8 per group). **P<0.01, ***P<0.001 by one-way ANOVA.

To explore the role of S100a9 in AD pathogenesis, we injected S100a9 lentiviral short hairpin RNA (sh-S100a9) or control lentiviral short hairpin RNA (sh-CTL) into the brains of 13-month-old Tg2576 and age-matched wild-type mice. Two months later, we evaluated learning and memory impairment in the Morris Water Maze task in sh-S100a9 or sh-CTL-injected mice. Tg2576 mice injected with sh-S100a9 (Tg_sh-S100a9) showed a significant difference from the Tg_sh-CTL group on the 6^th^ day of the learning sessions (p = 0.0055, F = 4.49; Figure 4A). We found no noticeable differences between the WT_sh-CTL and WT_sh-S100a9 groups (Fig. 4A). To confirm that the memory impairment in Tg2576 mice was actually improved by sh-S100a9 injection, we performed the probe test 48 h after the final trial and recorded the duration of time spent in zone 4 without the platform. Similar to the WT group, Tg_Sh-S100a9 mice stayed significantly longer in zone 4 than in the other zones (zones 1–3) (Figure 4B). However, there was no significant difference for Tg_sh-CTL mice in terms of time spent in different zones, and no noticeable difference in the WT mice treated with sh-S100a9 or sh-CTL (Figure 4B). These data show that S100a9 knockdown increased the spatial reference memory in Tg2576 mice.

### Treatment with sh-S100A9 Reduced the Number of Amyloid Plaques and Eosinophilic Pyknotic Neurons in Tg2576 Mice Brains

**Figure 5:**
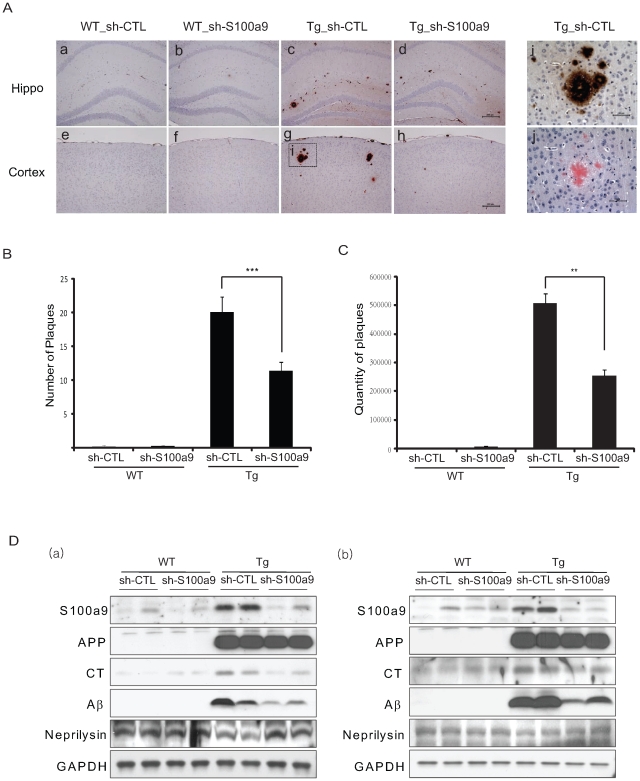
S100a9 knockdown reduced the number of amyloid plaques in Tg2576 mouse brains. At 2 months after lentiviral infection, immunostaining and western blot analyses were performed. (A) Immunostaining with 6E10 antibody for the detection of amyloid plaque in the mouse brain of Hippocampus (Hippo) and Cortex. Higher-magnification view of box in (g) is shown in (i). (j) is Congo red stained image of (i). Scale bars represent 200 µm in (d&h) and 50 µm in (i&j). (B) The number of plaques was counted in whole brain of each group (n = 10). ***P <0.001 by Student's *t-* test (C) Size and density of plaques was measured by Multi-Gauge program (version 3.0) in whole brain of each group (n = 10). Results are presented as the means of (QL-BG) ± SEM. **P<0.01 by Student's *t-* test. (D) Western blot analysis was performed with total lysates from the cortical region (a) or hippocampus region (b) of the brains in each group using C9 for APP and CT, 6E10 for Aβ, anti-Neprilysin or anti-GAPDH antibody. All data represent the means ± SEM from at least five independent experiments.

To investigate possible links between the severities of memory impairment and amyloid deposition, we examined amyloid plaque load and the protein levels of Aβ and CT in the brains of 15-month-old WT_sh-CTL, WT_sh-S100a9, Tg_sh-CTL and Tg_sh-S100a9 mice (i.e., after behavioral tests) using 6E10 antibody, which specifically recognizes amino acids 1–17 of the Aβ region. In the brain sections of Tg_sh-CTL mice, amyloid plaques were highly stained by 6E10 antibody in the cortex and hippocampal region (Figure 5A). Dense-cored plaques of amyloid were detected in brain sections of Tg_sh-CTL mice. However, the number of amyloid plaques in Tg_sh-S100a9 mice was significantly reduced (from 20.15±2.24 to 11.4±1.34, P = 0.001, Student's *t*-test; Figure 5B). In the brains of WT mice, few or no amyloid plaques were observed (Figures 5A&B). We then confirmed these data by quantifying the size and density of each amyloid plaque (Figure 5C). For detection of amyloid plaques, Congo red staining was also performed and Congo-red stained plaques concurred with Aβ immunoreactive plaques (Figure 5Aj).

Serial brain sections were also labeled with S100a9 antibodies. Lentiviral sh-S100a9 significantly reduced S100a9 expression in the brains of Tg_sh-S100a9 mice (data not shown). In both the cortex and hippocampus from mouse brains in each group, S100a9 expression was increased in the Tg_sh-CTL group and significantly decreased in the Tg_sh-S100a9 group compared with that in WT mice (figure 5D). There was no noticeable difference between WT_sh-CTL and WT_sh-S100a9 animals (Figure 5D).

The expression of APP was not changed between Tg_sh-S100a9 and Tg_sh-CTL mice, but, interestingly, the levels of Aβ and CT were decreased in the Tg_sh-S100a9 (Figure 5D), consistent with the decreased number of amyloid plaques (Figures 5A–C). These results suggest that S100a9 might be a contributing factor to increase Aβ and CT in Tg2576 mice. Therefore, we determined the effect of S100a9 on neprilysin, the Aβ-degrading enzyme, and measured the expression of neprilysin in the brains of each group. Neprilysin levels in Tg2576 mice were significantly reduced to about 40% of that in age-matched WT mice. However, neprilysin levels in Tg_sh-S100a9 mice were similar to those of WT mice (Figure 5D).

Eosinophilic pyknotic neurons suggesting neuronal degenerative changes were observed by hematoxylin and eosin (H&E) staining. In the serial brain sections of Tg_sh-S100a9 mice, the number of eosinophilic pyknotic neurons was reduced in cortex and hippocampal regions (CA3, DG) of the brain compared with that in the brains of Tg_sh-CTL mice (Figure S4). However, few eosinophilic pyknotic neurons were observed in the comparative regions of the WT_sh-CTL and WT_sh-S100a9 groups. Thus, these experiments indicate that S100a9 might be related to neurodegeneration in the AD animal model, Tg2576 mice.

## Discussion

The induction of the S100a9 gene was found in the brains of AD patients and AD animal models, Tg2576 and CT-Tg mice. From these results, we concluded that S100a9 might be involved in the pathogenesis of AD. Recently, S100a9 has been proposed to participate in the inflammation of AD pathogenesis [16], but the detailed molecular mechanism of these pathological events remains unknown. Therefore, we focused on the role of S100a9 in the neuroinflammation related to AD.

In this study, prominent staining of S100a9 was detected in the microglia of CT-Tg mice. In BV2 cells transfected with either CT50 or CT99 or treated with either Aβ or CT, the induction of S100a9 was confirmed at the mRNA and protein levels. In the luciferase promoter assay, the promoter of S100a9 gene was upregulated by CT. These results indicate that both Aβ and CT significantly induce S100a9 expression by direct or indirect regulation of S100a9 promoter activity. A number of distinct regulatory regions and binding partners of S100a9 have been verified in the nervous system [2], [26], [27]. In particular, C/EBPα and C/EBPβ have been shown to bind 1 kb upstream of S100a9 promoter, leading to the transcription of the S100a9 gene [27]. Tip60, which is one of transcription factor for the transregulation of CT [21], [28], has been proposed to be a co-activator of C/EBPα [29], suggesting that CT might induce S100a9 expression through interactions with Tip60 and C/EBPα in the nucleus. Although Aβ cannot activate gene transcription through nuclear translocation and direct binding to the transcription factor, Aβ may induce S100a9 transcription through activation or suppression of signaling molecules such as CREB (cAMP response element binding protein), BDNF (brain-derived neurotrophic factor) or CaN (calcineurin). Aβ has been reported to promote CaN-dependent CREB dephosphorylation or suppress the activation of BDNF, interfering with neuronal function and contributing to cognitive deficit in AD before the onset of massive neuronal degeneration [30], [31].

In calcium imaging assays, we found that Aβ or CT significantly increased [Ca^2+^]_i_ levels in BV2 cells, while silencing of the S100a9 gene reduced the increased [Ca^2+^]_i_ levels by Aβ or CT to the control levels. We hypothesized that Aβ or CT increased the expression of S100a9, leading increased [Ca^2+^]_i_ levels [24]. Microglia from AD brains had a higher [Ca^2+^]_i_ level than control. This elevated [Ca^2+^]_i_ level proves some executive functions of activated microglia in the pathogenesis of AD [25]. Activated microglia can proliferate, actively migrate to the site of injury, and release a variety of factors such as cytokines, chemokines, NO, and growth factors to affect the pathologic processes [32]. Here, we show that either Aβ or CT treatment induced significant increases of pro-inflammatory cytokines, IL-1β, TNF-α and iNOS in BV2 cells. But, treatment with si-S100a9 attenuated the increase of IL-1β, TNF-α and iNOS by CT. The induction of NO by CT was also greatly reduced by si-S100a9 treatment, suggesting that S100a9 is critical for the inflammatory response induced by CT. Our results suggest that S100a9 might induce the neuroinflammation by increasing the [Ca^2+^]_i_ level, although it cannot be excluded that S100a9 might affect both processes independently. We investigated the levels of other specific calcium-binding proteins, calbindin D-28K and other S100 proteins, S100a8 and S100B in Tg2576 mice brains. Our data indicate that calbindin D-28K, S100a8 and S100B are not induced in the Tg2576 mice brains compared with those in the wild type mice brains (Figure S5). And also there is no change in their levels between Tg_sh-CTL group and Tg_sh-S100a9 group (Figure S5). We assume that calcium dyshomeostasis are mainly induced by S100a9.

Here, we provide *in vivo* evidence that the up-regulation of S100a9 in the brains of Alzheimer's patients and an animal model of AD might be related to the pathogenesis of AD. Increased S100a9 expression might contribute to the impairment in synaptic plasticity and cognitive function as well as neurodegeneration. In this study, S100a9 knockdown was performed using S100a9 lentiviral short hairpin RNA in the brains of 13-month-old Tg2576 mice and age-matched wild-type mice. Two months after injection, we observed reduced S100a9 expression in both the cortex and hippocampus in the brains of Tg_sh-S100a9 mice. The Morris water maze task clearly showed that S100a9 knockdown restored the learning and memory function in Tg2576 mice. Memory dysfunction was severe in the Tg_sh-CTL group. In a previous study of Tg2576 mice, spatial memory loss coincided with the appearance of aggregated Aβ [22], and diffuse plaques, and biochemically extracted Aβ42 and Aβ40 were increased to levels like those observed in AD brains [33]. The presence of amyloid plaques containing predominantly Aβ and neurofibrillary tangles is believed to be a pathological feature of AD [1], [18]. Our data showed that S100a9 knockdown markedly reduced the number of amyloid plaques in Tg2576 mice brain. We also found that the levels of Aβ and CT were decreased in the brains of Tg_sh-S100a9 mice compared with those in Tg_sh-CTL mice.

A recent report showed that microglia in the brains of aged AD mice became dysfunctional in terms of Aβ clearance, and expressions of their Aβ-binding receptors and Aβ-degrading enzymes were significantly reduced; however, their ability to produce proinflammatory cytokines was maintained [34]. In our results, the level of the Aβ-degrading enzyme, neprilysin, was decreased in the brains of Tg2576 mice. However, S100a9 knockdown recovered the level of neprilysin to that of WT mice. These results suggest that S100a9 might play a role in APP metabolism by down-regulating neprilysin in the brains of Tg2576 mice. We also suggest that overproduction of Aβ or CT induced by an increase of S100a9 might accelerate neuronal degeneration. In fact, we found that the number of eosinophilic neurons in hippocampus was increased in the brains of Tg2576 mice, but was significantly reduced in Tg_sh-S100a9 mice. These findings strongly suggest that an increase in S100a9 may be closely related to the pathogenesis of AD.

Altogether, we demonstrate with these data that S100a9 is induced by either Aβ or CT, leading to the pro-inflammatory response and resulting in neuronal death. Also, S100a9 might be responsible for the production and accumulation of CT/Aβ through the regulatory protein like neprilysin in the brains of Tg2576 mice. Finally, S100a9 gene silencing significantly reduced AD pathology, including the number of plaques, and improved the learning ability of Tg2576 mice. Therefore, inhibition of S100a9 may be an alternative AD therapeutic design.

## Materials and Methods

### Sample Preparation and Microarray Analysis

We used brains of the transgenic mice (11months) model expressing the familial Alzheimer's disease (FAD) as making V717I (valine to isoleucine) ‘London’ mutation within an APP-CT99 which encompasses the β amyloid (Aβ) sequence. APPV717I-CT100 Tg mice were provided from Dr. Emson PC at Babraham institute. We extracted total RNA of hippocampus in three brains of 11 month-old APP_V717I_-CT100 Tg mice. Total RNA was prepared from using Trizol reagent (invitogen). All the microarray experiments were done with CodeLink Twinchip™ Mouse-20K (Amersham Bioscience) from Digital Genomics (Seoul, South Korea). Processed slides were scanned using an Axon GenePix 4000B Scanner with the laser set to 635 nm, the photomultiplier tube (PMT) voltage to 600 and the scan resolution to 10 µm. Images for each slide were analyzed using the CodeLink™ Expression Analysis Software v4.1(GE Healthcare Life Science). Genes showing significant expression changes (>2 fold) were selected with a cutoff of 5% of a q value. The Microarray dataset are deposited at Gene Expression Omnibus (GEO) database and are accessible through accession number (GSE18762).

### Human AD Brains

Paraffin-embedded brain stocks and the frozen tissues of from 69 to 87 years old-AD and age-matched control subjects were obtained from the Netherlands Brain Bank (NBB). In AD tissues, the neuropathological diagnosis is Alzheimer's disease (AD) and Braak & Braak stage V or VI. The neuropathological diagnosis of control is non-demented control and Braak & Braak stage 0 or 1. Coronal sections (4 µm) were cut through the hippocampus and processed for immunohistofluorescence. For western blot analysis, we used the frozen tissues of the brain. All experimental procedures were performed in accordance with ‘the Guidelines of the Ethics Committee at Seoul National University’.

### Cell Culture

The BV2, immortalized murine microglial cell line, was cultured in Dulbecco's modified Eagle's medium (DMEM; Life Technology) supplemented with 5% fetal bovine serum (Hyclone) and penicillin/streptomycin (100 U/ml/100 µg/ml) (Life Technology) at 37°C and 5% CO_2_

### RT-PCR

Total RNA (2 µg) isolated from brain tissues or cells with Trizol reagent (invitrogen) was used for cDNA synthesis by AccuPower RT premix (Bioneer). The abundance of transcripts in cDNA samples was measured by RT-PCR with primer as follows: S100a9 forward, 5′- CAGCATAACCACCATCATCG-3′, reverse, 5′-GTCCTGGTTTGTGTCCAGGT-3′; actin forward, 5′-CCAGATCATGTTTGAGACCT-3′, reverse, 5′-GTTGCCAATAGTGATGACCT-3′. The PCR reactions were subjected to 40 cycles of PCR amplification (95°C for 1 min, 58°C for 1 min, 72°C for 1 min). All results were normalized to actin.

### Antibodies

Anti-APP C-terminal polyclonal antibody (C9) was purchased from Chemicon (California); 6E10 (Chemicon), CD11b (Chemicon), IL-1β (R&D), TNF-α (R&D), iNOS (Santa Cruz), anti-S100a9 (R&D), anti-GAPDH (Santa Cruz), anti-tubulin (Santa Cruz), Neprilysin (alpha diagnostic).

### Western Blot Analysis

Cells or tissues were washed with phosphate buffered saline (PBS) and lysed in RIPA buffer with cocktail of protease inhibitors (Roche). Proteins were separated by SDS-PAGE and transferred to a PVDF membrane. The PVDF membrane was blocked with 5% nonfat dry milk in Tris-buffered saline. After 1 h blocking, the protein blot was confirmed with appropriate antibodies and detected using horseradish peroxidase-conjugated secondary antibody (Amersham Pharmacia).

### DNA Construct and Transfection

The CT50 and CT99 constructs in these experiments were previously reported in Chang et al.[19]. Briefly, CT50 or CT99 to encompass the last 50 or 99 amino-acid residues of human APP695 cDNA was subcloned into a pcDNA3-flag vector with the flag at the N-terminus. Cells were transiently transfected with individual constructs by using Fugene 6 according to the manufacturer's instruction (Roche, Germany).

### Luciferase Assay

Human S100a9 promoter in pGL3 vector provided from Dr. Claus Kerkhoff at university of Muenstet in Germany were cotransfected with CT, wtAPP or sweAPP in pcDNA vector and fe65 into SHSY5Y cells. After 48 h transfected, the cells were lysed by Luciferase Assay system with reporter lysis buffer (Promega, WI, USA). Luciferase activity was measured using a Biocounter M1500 luminometer (Lumac, GE Groningen, Netherlands). Protein concentrations were determined using Bradford protein assay reagent (Bio-Rad), and luciferase activities were normalized versus the obtained protein concentrations.

### Preparation and Treatment of CT and Aβ Peptide

The expression plasmid pET28a-CT99 tagging His was constructed by ligating CT99 digested with NdeI and BamHI into pET28a and transformed into Escherichia Coli. CT99 (CT) peptide was purified by Ni-NTA superflow (Qiagen) and then dialyzed against 10 mM Tris-HCl (pH 7.4), followed by lyophilization. We used synthetic Aβ_1–42_ (Aβ) peptide with >95% pure by RPHPLC chromatography (Sigma). Aβ peptide was dissolved to 1 mmol/L in 100% hexafluoroisopropanol (Sigma). Hexafluoroisopropanol was removed under vacuum, and the peptide was stored at −20°C. For the aggregation, the peptide was first resuspended in dry dimethylsulfoxide (DMSO; Sigma) to 5 mmol/L. For oligomeric conditions, F-12 (without phenol red) culture medium was added to bring the peptide to a final concentration of 200 µmol/L, and the peptide was incubated at 4°C for 24 h[35].

### Calcium Level Measurement Using Fluo-3/AM

BV2 cells were plated and cultured on glass coverslips coated with 10 mg/ml PEI (Polyethylenimine). The cells, pretreated with various dose of CT and Aβ peptide, were washed twice with Hank's solution (Gibco) and incubated for 20 min at 37°C in the same buffer containing 10 µM Fluo-3/AM. After mounting the cells, its images were obtained using a laser-scanning confocal microscope (Zeiss). Excitation was achieved using an Argon ion laser (wavelength (λ)  = 488 nm) and fluorescence was measured at λ>515 nm. Changes of [Ca^2+^]i levels were measured using relative fluorescence compared with that of control group.

### siRNA Treatment

A siRNA oligonucleotide targeting the mouse S100a9 mRNA (si-S100a9) was designed from Qiagen. BV2 cells were seeded in six-well plates at 2×10^5^ cells/well and incubated overnight at 37°C. Then cells were transfected with 20 nM of siRNA using HiPerFect (Qiagen) as directed by the manufacturer. We treated CT peptide at 12 h post transfection siRNA. Cells were incubated for 48 h after transfecting siRNA.

### Assay for Nitric Oxide (NO) Derivatives

NO_2_ accumulation in the medium was used as an indicator of NO production. Nitric oxide induced by CT was measured by Griess reagent (G4410, Sigma). After the appropriate incubation time with the peptides on the BV2 cell, supernatant was harvested, and then mixed with equal volumes of 1xGreiss Reagent and sample (working range: 0.43∼0.65 µmolar nitrite) in the 96well plate. Spectrophotometric absorbance was measured at 540 nm after 5 min. The values measured were compared with the vehicle control.

### Immunohistochemistry

Mice brains and human AD brains in 10% neutral buffered formalin for 48 h were dehydrated and embedded in paraffin. Before immunostaining, slides were deparaffinized in xylene and then dehydrated through graded alcohols to water. The fluorescent immunohistochemistry was performed with appropriate primary antibodies at 4°C for O/N and visualized using Cy3-conjugated or FITC-conjugated secondary antibody (Jackson, West Grove, Pennsylvania). DAPI counter staining was performed. Images were collected using the LSM 510 program on a Zeiss confocal microscope (Carl Zeiss MicroImaging, Inc.).

For the non-fluorecence labeling, Immunohistochemistry was performed using a Vectastain avidin-biotin complex (ABC) elite kit. Reaction product was detected using 3,3-diaminobenzidinetetrahydrochloride (DAB). Photomicrographs were acquired with a color digital camera DFC280 (Leica) attached to a microscope (BX-51; Olympus).

### sh-S100a9 Virus Preparation and Production

The sh-S100a9 hairpin oligo (5′-CCGGGCTGAGCTTTGAGGAGTGTATCTCGAG ATATCACTCCTCAAAGCTCAGCTTTTTG-3′; Clone ID: NM_009114.1-255s1c1) was selected from five Lentiviral transfer vector of the sh-S100a9 containing mouse U6 promoter received in bacterial Glycerol Stock by Sigma-Aldrich (Missouri, USA). The transfer vector plasmid (sh-CTL or sh-S100a9), together with the packaging construct plasmid pCMVΔR8.2 and the envelope plasmid pVSV-G, were cotransfected into HEK293T cells to produce the viral particles. After overnight incubation, the medium was changed. Supernatants were collected from day 2 to 4 post transfection. The viral particles in the supernatant were filtered through a 0.45 µm pore size filter and then sediment by ultracentrifugation at 35,000 rpm, 4°C for 2 h. The pellets were re-dissolved in PBS. For in vivo experiments, the virus was further centrifuged for 50,000 rpm 4°C for 2 h and re-dissolved in PBS, resulting in a final 1,000-fold concentration.

### Animal Preparation and Injection of sh-S100a9 into Tg2576 Mice Brains

APPswe Tg2576 mice were obtained from Taconic Farms (Germantown, NY) and were bred by mating male mice with C57B16/SJL F1 females, as recommended by the suppliers and as described by others [33]. Studies were performed by comparing 13 month-aged heterozygous transgenic (Tg2576) mice from to age-matched transgene-negative littermates (wild type). Mice were divided into four groups (8 mice per each group) and each anaesthetized mouse in WT or Tg group was received 2 µl of the concentrated sh-CTL or sh-S100a9 virus, respectively using a Kopf stereotaxic frame (Kopf Instruments, Tujunga, CA), loaded into the hippocampus (AP, 0.18 mm; ML, 0.20 mm; DV, 0.19 mm) over a period of 5 min and retracted after an additional 5 min. The procedures were overseen by the Animal Care and Use Committee of Seoul National University.

### Morris Water Maze Task

The Morris water maze was performed at 2 months after virus injection. The experimental apparatus consisted of a circular water tank (diameter = 140 cm; height = 45 cm), containing water at 23°C to a depth of 35.5 cm and rendered opaque by adding milk. A platform (diameter = 15 cm; height = 35 cm) was submerged 1 cm below the water surface and placed at the midpoint of one quadrant. The pool was located in a test room containing various prominent visual cues. Three training trials per day were conducted for 9 consecutive days, with a rotation order per trial in a group. Mice were placed in the pool at one of three quadrant starting positions (except one quadrant containing platform). In each training trial, the time required to escape onto the hidden platform was recorded. Mice that found the platform were allowed to remain on the platform for 30 s and then were returned to the home cage during the inter-trial interval. Mice that did not find the platform within 60 s were placed on the platform for 30 s at the end of trial. After 48 h of the final trial, a single probe trial was conducted. The escape platform was removed, and each mouse was allowed to swim for 60 s in the maze. The time spent in the quadrant that previously contained the platform was recorded as the total time in the pool.

### Congo Red Staining

Deparaffinized and hydrated sections were incubated in a freshly prepared alkaline alcoholic saturated sodium chloride reagent (2.5 mM NaOH in 80% reagent-grade alcohol) for 20 min at room temperature and then were incubated in 0.4% Congo red (W/V, Sigma) in an alkaline alcoholic saturated sodium chloride reagent (freshly prepared and filtered just prior to use) for 30 min at room temperature. Sections were washed in distilled water and counterstained with hematoxylin for 1 min. And then sections were rinsed through ascending grades of ethanol with a final three changes of 100% reagent-grade ethanol, cleared in xylene and cover slipped with permount (Fisher Scientific).

### Hematoxylin and Eosin Staining

Slides were incubated with Mayer's hematoxylin (DakoCytomation) and eosin (Acros organics). Sections were dehydrated through graded alcohols, cleared in xylene, and cover slipped in Canadian balsam solution.

### Statistical Analysis

Statistical analysis was performed by Student's *t*-test and ANOVA using an SASS program (SPSS Inc., Chicago, Ill) to study the relationship between the different variables. Values of *P*<0.05 were considered to indicate statistical significance.

## Supporting Information

Figure S1 S100a9 was induced in the brains of Human AD patients. (A) The level of S100a9 protein was examined in the hippocampus of AD patients' brains. Tubulin was used as a loading control. This is a representative blot (n = 10). (B) We quantified the density of S100a9 with gel doc and normalized it to that of Tubulin (n = 10). *P<0.05 by Student's t-test.
(0.85 MB EPS)

Figure S2 [Ca2+]i level induced by Aβ or CT in a dose-dependent manner. (A) BV2 cells grown on glass coverslip for 48 h were treated with 0.1, 1, or 10 µM CT and 1 or 10 µM Aβ and then [Ca2+]i level was measured by Fluo3 AM. Scale bars represent 50µm. (B) Graph shows the fold-change of relative fluorescence intensity versus that of control. (n = 10). Results are presented as the means ± SEM. NC: negative control, **P<0.01, ***P <0.001 by Student's t-test.
(3.68 MB EPS)

Figure S3 S100a9 induced by Aβ increased [Ca2+]i level in BV2 cells. (A) [Ca2+]i mages obtained by Fluo3 AM at 48 h after treatment with 10 µM Aβ and 20 nM si-S100a9 in BV2 cells. Scale bars represent 50µm. si-CTL is a control containing the scrambled sequence of S100a9 (B) The histogram shows the ratio of [Ca2+]i levels to NC group. [Ca2+]i levels in BV2 cells treated with Aβ and si-S100a9 was compared to that in BV2 cells with Aβ and si-CTL (control) (n = 5). Results are presented as the means ± SEM. NC: negative control, **P <0.01 by Student's t-test.
(2.34 MB EPS)

Figure S4 S100a9 knockdown reduced the number of eosinophilic cells in Tg2576 mice brains. At 2 months after lentiviral infection, Hematoxylin and eosin (H&E) straining was performed in hippocampus and cortex. Scale bars indicate 100 µm in large square box and 20 µm in small square box.
(2.42 MB EPS)

Figure S5 Expression of calcium binding proteins in Tg2576 mouse brains. At 2 months after lentiviral infection, western blot analysis was performed with total lysates from the cortical region of the brains in each group using anti-Calbindin D-28K, anti-S100a8 and anti-S100B antibody. The membrane was stripped and reprobed with GAPDH to confirm equal loading. This is a representative blot from at least five independent experiments.
(1.19 MB EPS)
